# Identification of Chinese Herbal Medicines with Electronic Nose Technology: Applications and Challenges

**DOI:** 10.3390/s17051073

**Published:** 2017-05-09

**Authors:** Huaying Zhou, Dehan Luo, Hamid GholamHosseini, Zhong Li, Jiafeng He

**Affiliations:** 1 School of Information Engineering, Guangdong University of Technology, Guangzhou 510006, China; zhhying1224@163.com (H.Z.); jfhe@gdut.edu.cn (J.H.); 2 College of Medical Information Engineering, Guangdong Pharmaceutical University, Guangzhou 510006, China; 3 School of Engineering, Computer and Mathematical Sciences, Auckland University of Technology, Private Bag 92006, Auckland 1142, New Zealand; hamid.gholamhosseini@aut.ac.nz; 4 College of Traditional Chinese Medicine, Guangdong Pharmaceutical University, Guangzhou 510006, China; lizhongyxy@126.com

**Keywords:** electronic nose, Chinese Herbal Medicines, odor identification, olfactory systems

## Abstract

This paper provides a review of the most recent works in machine olfaction as applied to the identification of Chinese Herbal Medicines (CHMs). Due to the wide variety of CHMs, the complexity of growing sources and the diverse specifications of herb components, the quality control of CHMs is a challenging issue. Much research has demonstrated that an electronic nose (E-nose) as an advanced machine olfaction system, can overcome this challenge through identification of the complex odors of CHMs. E-nose technology, with better usability, high sensitivity, real-time detection and non-destructive features has shown better performance in comparison with other analytical techniques such as gas chromatography-mass spectrometry (GC-MS). Although there has been immense development of E-nose techniques in other applications, there are limited reports on the application of E-noses for the quality control of CHMs. The aim of current study is to review practical implementation and advantages of E-noses for robust and effective odor identification of CHMs. It covers the use of E-nose technology to study the effects of growing regions, identification methods, production procedures and storage time on CHMs. Moreover, the challenges and applications of E-nose for CHM identification are investigated. Based on the advancement in E-nose technology, odor may become a new quantitative index for quality control of CHMs and drug discovery. It was also found that more research could be done in the area of odor standardization and odor reproduction for remote sensing.

## 1. Introduction

As the basis of traditional Chinese medicine, Chinese Herbal Medicines (CHMs) have been used for the treatment and prevention of human diseases for thousands of years in China. A unique systematic approach towards the use of CHMs has been developed through long-term practice and CHMs have shown excellent medical effectiveness with minimal side effects [1,2]. Furthermore, the important role of CHMs in the prevention and treatment of many epidemic, chronic and infectious diseases has been widely demonstrated and recognized by the international community [1]. The latest statistics reveal that the number of kinds of CHMs is more than 12,800 [3]. In 2014, there were 3813 Chinese medicine production enterprises, with revenue of up to 730,200 million Yuan and distribution in 183 countries and regions [4,5]. However, due to the wide variety, complex sources and diverse specifications of the herb components of CHMs, occurrences of fake and poor quality products have emerged in the CHMs market. This has seriously restricted the use of CHMs and the related international market [6]. Therefore, the study of effective methods for quality control of CHMs has become an urgent issue.

Odor is one of the most important properties of CHMs. Many CHMs such as *Citrus reticulata* Blanco (Chenpi) or *Alpinia officinarum *Hance (Gaoliangjiang) have different strong odors coming from their respective volatile components, and moreover, the same kind of CHM can release distinct odors due to different quality, growing region or storage time [4,7]. Many researchers have investigated methods for assessing quality of CHMs based on odor of their volatile components [6,8]. Zhang [9] reported that smell is an important evaluation index in traditional quality evaluation of CHMs. The smell of CHMs is directly related to their chemical composition, which is the interface between the external characteristics and intrinsic quality of CHMs. For example, raw *Amomum villosum *Lour. (Sharen) has a strong aromatic smell, indicating its good quality and excellent effect in its associated medical treatment. A change of smell indicates that the quality of *Amomum villosum *Lour. is also changed [6,10].

The identification of CHMs has traditionally been based on methods such as physico-chemical analysis, gas chromatography-mass spectrometry (GC-MS) technology, spectroscopic techniques and microscopic methods. These methods are complicated, time-consuming and costly, and generally they cannot satisfy the increasing requirements for the real-time detection and identification. Furthermore, the human olfactory system, as a smell assessment instrument, is subjective, with low accuracy and limited functionality [11]. Consequently, there is a considerable need for an instrument that can mimic the human nose to identify different kinds of CHMs, and make the identification more objective and accurate. The electronic nose (E-nose) is an instrument mimicking the sense of smell for detecting and identifying complex odors by using an array of embedded gas sensors [12,13,14]. When the sensor array is influenced by an odor stimulus, it generates a smell print, which is the response of the sensor array and represents an electronic fingerprint characteristic of each sample [13]. These smell prints form a recognition pattern for qualitative analysis using an appropriate multivariate tool. The E-nose with real-time information processing characteristics greatly facilitates fast monitoring of volatile components of diverse molecular structure [15,16,17,18].

## 2. Design of the Study

Recent journal papers mainly published since 2011 were selected for this review. We chose some keywords like E-nose, electronic nose, olfaction technology, identification of Chinese Herbal Medicines and odor detection to search for the related journal papers in the Google Scholar, China National Knowledge Internet (CNKI), IEEE Xplore, ScienceDirect and SpringerLink databases. We thus found about 19,400 papers. We then limited the number of papers to 126 based on the following criteria: Duplicate articles were eliminated and some additional records were excluded after reviewing the individual titles and abstracts. Next, we reviewed the included studies and evaluated the full-text articles (journal articles and conference proceedings) for eligibility. We excluded those articles that were not considered original research, such as letters to the editor and comments. Because this review paper is focused on the application of E-noses for identification of CHMs, we excluded studies that solely employed physico-chemical characteristics or microscopic observation for the identification of CHMs.

## 3. Electronic Nose (E-Nose)

**Figure 1:**
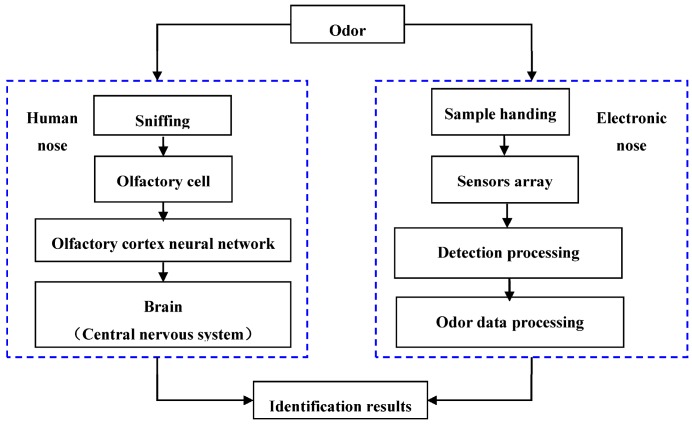
Comparison of human olfactory system and electronic nose.

The E-nose, as a machine olfaction system, can mimic the human nose and perform complex pattern recognition like the human olfactory system. It is composed of three parts: (1) a sample handling system; (2) a detection system that is made up of an array of gas sensors with partial specificity; and (3) an odor data processing system [16,19]. A comparison of the E-nose and the human nose with respect to their main functional blocks is shown in Figure 1. The E-nose instrument can detect the presence of VOCs of diverse molecular structures with high accuracy and reliability regardless if it smells more or less. Furthermore, the E-nose may accomplish qualitative and quantitative analysis of odor samples [20,21]. Compared to traditional odor analysis methods, such as GC-MS and Fourier transform infrared (FT-IR) spectrometry, E-nose is an easy built system that possesses simple sample pretreatment, non-destructive features, relatively fast assessment detection, a wide odor operating range, and generally high sensitivity and selectivity to the tested odorants. Therefore, E-noses have been widely used in medicine and health care [22,23,24,25], agriculture and food products [18,20,26,27,28,29,30,31,32,33,34,35], public security [36] and environmental monitoring [16,17]. Since 1993, the number of publications in the area of electronic olfaction has reached a record of more than 12,000 articles [13]. However, reports or publications on the application of E-nose systems in quality control of CHMs have been limited [1,7]. Table 1 provides a list of E-nose models for commercial and non-commercial applications [13].

### 3.1. Sample Handling System

Sample handling to transfer volatile aromatic molecules from the source to the E-nose sensor array is a critical step that affects the data analysis performance of an E-nose [37]. Before sample detection, some parameters, such as sample temperature, equilibration time, vial size and sample quantity should to be optimized [13]. In addition, due to the poor repeatability of the manual headspace injection of an E-nose system, an automatic headspace sampler is usually applied. Thus the smell of volatile components is transferred to the sensor array by a constant flow of an inert gas. In order to ensure the smell of volatile components of the sample present in the headspace flow into the sensor array at a steady speed, some common sampling methods such as static headspace (SHS), purge and trap (P&T) and dynamic headspace (DHS), solid-phase micro-extraction (SPME), stir bar sorptive extraction (SBSE) and inside-needle dynamic extraction (INDEX) are used [38,39].

### 3.2. Detection System—Gas Sensor Array

The main part of the E-nose detection system is the gas sensor array, which is composed of a number of sensors with a broad spectrum of response characteristics and large cross sensitivity to different gases. These gas sensors can convert gas concentrations into electrical signals, which are essential components of an E-nose to accomplish the qualitative or quantitative analysis of the monitored samples. The key design criteria are to select a suitable gas sensor array to improve the performance of the E-nose system in monitoring the selected samples [37]. As shown in Table 2 [16,27], there are several commonly used gas sensors. Among them, MOS and CP are considered as the most popular sensors due to their low cost, fast response, high sensitivity and ability to detect a large number of gases [13,40,41].

Currently, researchers have shown more interest in studying novel gas sensors composed of new materials, such as olfactory receptors of animals, organic field-effect transistors, molecularly imprinted polymers and nanomaterials [40,48,49,50]. For example, in a bioelectronic nose, the sensitive materials are usually made of animal olfactory receptor, cell or tissue. Volatile chemicals are detected and identified using optical, electrochemical and acoustic wave detection devices due to their advanced rapid response time, high sensitivity and good selectivity features. Liu [51] designed a bionic E-nose using chemical receptor protein Ac-ASP3 of bee as the sensitive material to detect and identify different concentrations of ligand molecules (isoamyl acetate). The results show that the bionic E-nose can accomplish quantitative analysis of volatile compounds [52,53]. However, bioelectronic noses have some disadvantages such as short lifetime, poor repeatability and difficult mass production process [42,43].

During the past decade, field-effect transistors (FETs) have been successfully used in modern electronics, computers and hand-held devices. Organic field-effect transistor (OFET)-based gas sensors have also attracted great interest due to their high selectivity, repeatable response and low-cost production [54]. Ayadi [46] proposed a novel technique to investigate gas sensitivity of materials for implementation in FET-based gas sensors. It was found that the surface charge induced by gas species adsorption, allows the estimation of maximum surface charge variation, which corresponds to the saturation of all possible adsorption sites. In particular, high-performance FET-based sensor integration with nanomaterials has demonstrated great prospects in sensing applications [54,55].

### 3.3. Data Processing System

#### 3.3.1. Odor Signal Pretreatment

The initial odor response of a gas sensor array often contains interference or noise. In order to reduce the complexity of the data processing, it is necessary to carry out signal denoising, filtering, data compression and normalization [56,57,58]. After the odor response has been smoothed, drift compensated, and outliers eliminated, the machine olfactory system then uses multivariate statistical signal processing methods to establish a characteristic odor pattern and form a characteristic odor map.

According to the characteristics of the output signal of the gas sensor array, different signal processing methods are adopted to improve the accuracy and linearity of the E-nose measurement. The purpose of the pretreatment is to extract relevant information and prepare the signal for the subsequent multivariate pattern recognition. Commonly used signal preprocessing techniques include baseline processing, data compression (feature selection or extraction) and normalization [11,59,60,61]. Some methods for baseline correction are listed in Table 3 and methods for data transformation are listed in Table 4.

Odor signal pretreatment has a great influence on the performance of an E-nose, as it not only reduces noise, complexity and recognition errors, but also improves the E-nose identification performance. At the same time, odor signal pretreatment also provides a standard odor data set for subsequent pattern recognition in the E-nose. 

#### 3.3.2. Feature Extraction

When the multivariate odor information is obtained by the sensor array, the odor data used as electronic fingerprints of the volatile components of CHMs, may contain irrelevant or redundant features [62]. Feature extraction techniques are intended to cope with the redundancy problem by selecting a subset of features that can facilitate data interpretation while reducing data storage requirements and improving prediction performance [63,64,65,66,67].

The commonly used feature extraction methods like independent component analysis (ICA), principal component analysis (PCA), linear discriminant analysis (LDA) and manifold learning (ML) are regarded as the most fundamental and powerful tools [68,69,70,71]. During the feature extraction (or selection), a smaller size of feature data preserving most of information in the raw data is extracted. The selected feature subset is considered as the “optimal” subset of features [72,73,74]. In this phase, the basic principle is to maximize the odor information contained in the new feature vector [75]. The feature extraction step, also called data dimensionality reduction, is essential to the performance of the subsequent classification stage.

#### 3.3.3. Pattern Recognition

After the feature extraction step and formation of an “optimal” subset of features, the second stage of data analysis system is pattern recognition. During this stage, a classifier can be designed to assign an unknown feature vector to one class from a previously trained set of feature vector labels. The classification results are visualized in the form of graphics, text, or tables. Moreover, the classification model is to be evaluated with additional data or validation dataset to estimate its accuracy [76,77,78].

Pattern recognition is a key part in the development of E-nose systems capable detecting, identifying or quantifying different volatile components. In the field of identification of CHMs, the selection of an appropriate pattern recognition method is crucial for the classification accuracy of an E-nose. The commonly used pattern recognition methods are usually divided into statistical pattern recognition and intelligent recognition modes [79,80,81]. These pattern recognition methods are summarized in Table 5 [82,83,84,85,86,87]. Among them, K-nearest Neighbor (KNN), Support Vector Machine (SVM) and Artificial Neural Network (ANN) are the most widely used.

## 4. E-Nose Applications in Identification of CHMs

**Figure 2:**
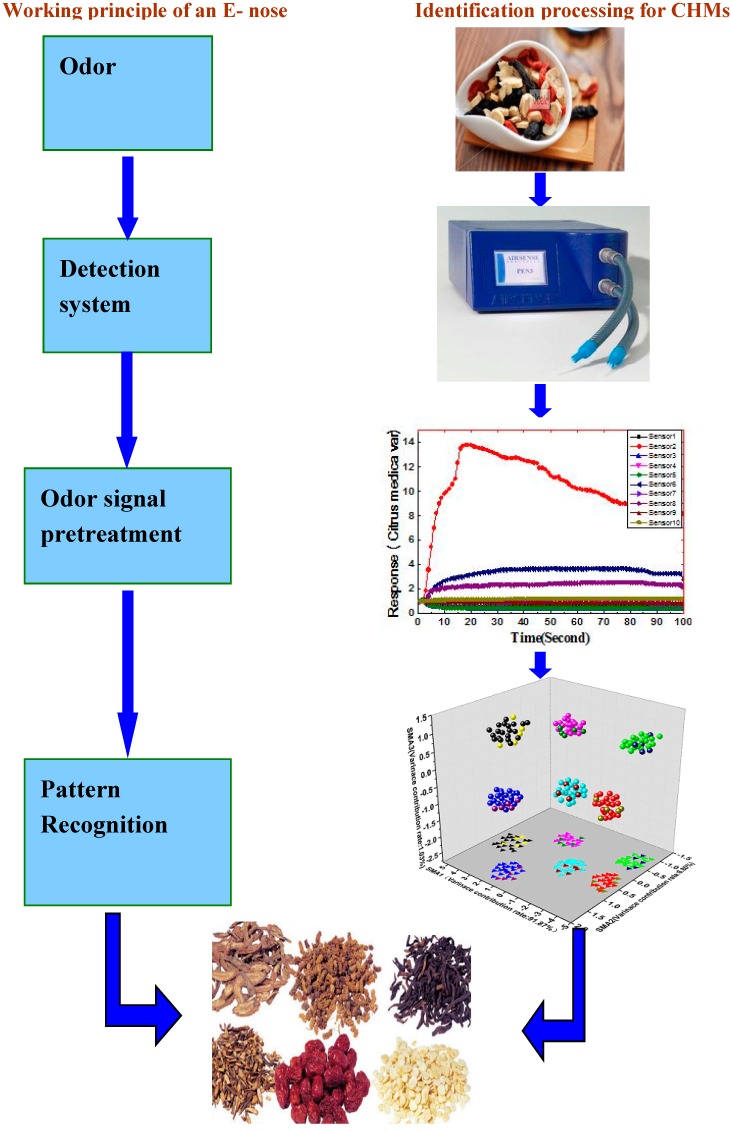
The working principle of an E-nose and the identification processing for CHMs.

Recently, with the continuous development of sensor technology and the improvement of pattern recognition methods, E-nose system technology is developing rapidly, and its application fields are expanding, ranging from environmental monitoring to medical applications. However, in the field of quality control of CHMs, there are limited publications on the application of E-nose technology. Since 2010, the number of publications on identification of CHMs based on E-noses is about 50 articles [88,89]. This demonstrates that the applications in identification of CHMs based on E-noses have begun to raise attention among researchers. Figure 2 shows the working principle of an E-nose and the process of identification of CHMs. The major findings related to the application of E-nose systems for identification of CHMs are presented in Table 6. In this section we provide a short description of E-nose system applications in the field of identification of CHMs focusing on four aspects: species identification, processed products identification, regional identification and storage time identification.

### 4.1. Species Identification of CHMs

In the CHMs market, it is very difficult for ordinary people to distinguish between CHMs which are similar in appearance. Therefore, herb species identification is an important part of the quality control in the traditional Chinese medicine market. Odor is a very important evaluation index of CHMs. Only experienced pharmacists can identify many commonly used CHMs with special fragrant smells, according to their odor characteristics. E-nose systems can simulate the human and animal olfactory function, and "smell" the whole odor of the CHMs, and then establish scent fingerprints by extracting information about the volatile components of CHMs [90]. Thus, the purpose of identification of different Chinese medicinal materials and verification of the authenticity of the CHMs is achieved [91,92].

Since many *Apiaceae* plants with antimicrobial activities have similar characteristics, it is difficult to distinguish them from each other. Lin [93] studied *Apiaceae* plants using an E-nose system composed of a MOS array and identified different kinds of *Apiaceae* plants using multivariate statistical analyses. The results showed that the response values were positively related to the different kinds of *Apiaceae* plants. According to the smell differences among samples, ten different kinds of *Apiaceae* plants could be classified correctly and the identification rate of ten-fold cross validation was 94.71%. Another study was performed using a portable PEN3 E-nose (Airsense Analytics, Schwerin, Germany) with PCA and LDA analysis methods to discriminate seven kinds of CHMs, *Illicium verum *Hook. (Bajiaohuixiang), *Amomi Fructus Rotundus* (Doukou)*, Ligusticum chuanxiong *hort. (Chuanxiong), *Eugenia caryophyllata *Thunb. (Dingxiang), *Schizonepeta tenuifolia *Briq. (Jingjie), *Cinnamomum cassia *Presl (Rougui), *Amomum villosum *Lour. (Sharen). In the PCA model, the cumulative variance contribution rate of the first two principal components was up to 99.338% and the first principal component variance contribution rate was 95.886%, while the the variance contribution rate of the second principal component was 3.452%; In the LDA model, the cumulative variance contribution rate of the first two principal components was 93.487% and the first principal variance contribution rate was 78.761%, while the variance contribution rate of the second principal axis was about 14.726%. At the same time, the predictive ability of the two models is evaluated by cross validation. The experimental results showed that the correct recognition rates were 96% and 98%, respectively [94]. Moreover, *Amomum villosum *Lour., *Pogostemon cablin (Blanco) *Benth. (Guanghuoxiang), *Leonurus japonicus *Houtt. (Yimucao), *Houttuynia cordata *Thunb. (Yuxingcao), *Mentha haplocalyx *Briq. (Bohe) and *Bupleurum chinense *DC. (Chaihu), the six kinds of typical CHMs in South of the Five Ridges were investigated using an E-nose system. According to the comparative study of odor fingerprints constructed based on the smell of the six CHMs, the number of common peaks and their values showed great differences. For example, the odor fingerprint of *Mentha haplocalyx *Briq. had 15 common peaks and the average value of these 15 common peaks was the largest, while the odor fingerprint of *Bupleurum chinense *DC. had only 11 common peaks and the average value of the 11 common peaks was the smallest. The research results showed that the E-nose system could “smell” the difference between the six kinds of samples and identify them [95].

### 4.2. Identification of Processed CHM Products

Raw CHMs and processed CHMs have different effects because of the changes of some components in the herbs during processing. Some processed CHMs, such as processed *Corydalis yanhusuo *W. T. Wang ex Z. Y. Su ex C. Y. Wu (Yanhusuo) and processed* Cyperus rotundus *Linn. (Xiangfu), have better effect for treating disease than the corresponding raw products. During processing, those ingredients having strong irritant properties and no discernable effect on the disease treatment can be removed, which makes the CHMs convenient to take for patients. Smell has thus also become an important factor to judge the quality of CHMs and distinguish between raw products and processed ones [96]. Shen [97] adopted an E-nose system to identify samples of natural *Atractylodes macrocephala *Koidz. (Baizhu) and processed *Atractylodes macrocephala *Koidz. PCA analysis was used to analyze the data, and the results showed that there was a significant difference in smell between the natural samples and the processed ones. Based on the analysis of odor fingerprints, the relative standard deviation (RSD) of the relative peak area of the common peaks were less than 1.2%, and the relative retention time of each peak was less than 1.1%, which showed that the method had good repeatability. In addition, the results were also confirmed by chemical composition analysis (using silicagel column chromatography). Xu [98] structured an E-nose system and differentiated four different samples of processed *Coptis chinensis *Franch. (Huanglian) based on the objective odor information combined with a statistical analysis method. The analyzed results showed that there were significant differences in odor characteristics among four different processed *Coptis chinensis *Franch. products. Furthermore, PCA analysis can more clearly distinguish the raw *Coptis chinensis *Franch. and different processed ones than the other three methods. At the same time, the initial discriminant rate and cross validation rate in PCA analysis were 100% and 94.4%, respectively. In this study, the different odor characteristics of raw *Coptis chinensis *Franch and processed *Coptis chinensis *Franch were identified by the E-nose system, combined with statistical analysis methods.

Huang [99] studied the raw and processed products of *Areca catechu *L. (Binglang), collected based on the color information by means of a color difference meter and odor information with the help of an E-nose system. He found that the characteristic color parameters and characteristic odor parameters, which were analyzed by employing PCA, LDA and ANN methods, were changed with the degree of stir frying of *Areca catechu *L,. This provides a new approach for the study of discrimination of processed CHM products.

### 4.3. Regional Identification of CHMs

Ecological and geographical factors, such as latitude, altitude, topography and geomorphology, affect the light, temperature, soil and precipitation and play a crucial role in the commercial growth of CHMs [100]. The same kind of CHM from different production areas may contain different volatile components, which affects the therapeutic effect of the CHM [101]. *Fritillaria cirrhosa *D. Don (Chuanbeimu) produced in Sichuan Province in China can moisten the lungs to stop coughs and is suitable for the treatment of cough Runfei and lung heat asthenia cough, whereas *Fritillaria thunbergii *Miq. (Zhebeimu) produced in Zhejiang Province in China has the effect of eliminating phlegm and Qingfei. It is suitable for the treatment of heat phlegm cough in patients [102]. Therefore, it is essential to identify CHMs according to their growing region. Using an E-nose system for such identification can be better and faster approach.

Li [103] studied the content and smell of Chinese* Panax ginseng *C.A. Mey. (Chinese Hongshen) and Korean* Panax ginseng *C.A. Mey. (Korean Renshen) by using an E-nose. He successfully identified these herbal medicines and confirmed the advantages of the E-nose system in such applications. In the three analysis methods used, the ten-fold cross validation rates were 96.12% for PCA, 97.56% for DFA and 92.39% for SIMCA. Chen [104] constructed an E-nose system to carry out the analysis of different *Ligusticum chuanxiong *hort. (Chuanxiong) samples coming from Aoping and Xudu in Sichuan. The results revealed that the volatile components of different grades of *Ligusticum chuanxiong *hort. from the same origin had similar properties, while the volatile ingredients of *Ligusticum chuanxiong *hort. From different growing areas had significant differences. The experimental results showed that the correct identification rate was 92.1% based on an E-nose system. Han’s research [105] showed that the combination of E-nose detection technology and chemical methods could be very sensitive for detecting the smell of white* Chrysanthemummorifolium* RaTnat. (Hangbaiju) from different regions. The cross validation rates were 94.38% for PCA and 91.46% for DFA. Zheng [106] studied a great number of batches of *Mentha haplocalyx *Briq. samples coming from Guangdong Province and Guangxi Province in China. He built odor fingerprints based on an E-nose system by using PCA and PLS models to analyze the odor information. The odor fingerprint of *Mentha haplocalyx *Briq. samples from Guangdong Province had 18 common peaks with an average value of 12.67, while *Mentha haplocalyx *Briq. from Guangxi Province had only 14 common peaks with an average value of 11.81. This proved that it was feasible to use scent fingerprint to distinguish between *Mentha haplocalyx *Briq. from different production areas. Zou [107] detected the odor of different kinds of *Amomum villosum *Lour. produced in different regions and also established their odor fingerprints based on an E-nose system. Among five selected analysis models, the performance of NBN was the best with an initial discriminant rate and cross validation rate of 98% and 95.2%, respectively. It confirmed that the MOS sensor array of an E-nose was able to identify different varieties of *Amomum villosum *Lour and also had the potential to analyze the chemical composition of *Amomum villosum *Lour. Lin’s [108] investigation of the odor response of *Oxybaphus himalaicus *Edgew. (XimalayaZimoli) showed that the odor of *Oxybaphus himalaicus *Edgew. from different areas was not the same. In this research, Discriminant Factorial Analysis (DFA), Hierarchical Cluster Analysis (HCA) and ANN methods were employed. The performance of ANN model was the best, and the initial discriminant rate and cross validation rate were 100% and 96.8%, respectively.

Wang [109] studied three kinds of CHMs—*Saposhnikovia divaricata* (Turcz.) Schischk. (Fangfeng), *Bupleurum chinense *DC. (Chaihu) and *Angelica sinensis *(Oliv.) Diels. (Danggui)—based on an E-nose system. The *Saposhnikovia divaricata* (Turcz.) Schischk. samples came from the Yunnan, Gansu and Henan provinces in China. The *Bupleurum chinense *DC. samples were from the Altai area, Mishima and Hailar, and the *Angelica sinensis *(Oliv.) Diels. samples came from Gansu Province and Yunnan Province. Three analysis models, PCA, SIMCA and DFA, were constructed and different feature extraction methods were also studied. The results revealed that the three kinds of CHMs could be effectively distinguished according to the odor characteristics of samples from different regions based on the three analysis models. A feature extraction method, based on the combination of the extreme value of the sensor response and the ratio between them, was the best of these models. In addition, the SIMCA model demonstrated more advantages than the other two in identification of the three CHMs. In SIMCA model, in identification of *Angelica sinensis *(Oliv.) Diels., the initial discriminant rate and ten-fold cross-validation rate were 96.6% and 95.2%, respectively. In the identification of *Bupleurum Chinense *DC., the initial discriminant rate and ten-fold cross-validation rate were 94.8% and 93.9%. In the identification of *Saposhnikovia divaricata* (Turcz.) Schischk., the initial discriminant rate and ten-fold cross-validation rate were 91.8% and 88.3%. 

### 4.4. Storage Time Identification of CHMs

Almost all CHMs have certain seasonal and regional characteristics. After harvesting, CHMs may be dried and stored for some time. The storage time may have a significant impact on the odor of CHMs. During the storage, the volatile components in many CHMs will gradually evaporate and the odor will change a lot. These changes affect the quality of the CHMs and may reduce the effectiveness of treatment [110,111]. For example, *Rheum officinale *Baill. (Dahuang) can lose 20~30% of the active ingredient if stored for more than a year, and if *Rheum officinale *Baill. is stored for more than five years, all of the active ingredient will be lost [112,113]. However, in the case of other CHMs, such as *Citrus reticulata* Blanco (Chenpi) and *Asini Corii Colla* (Ejiao), in order to achieve the better treatment effect, the herbs need to be stored for more than five years [113].

Therefore, in order to accurately identify the quality of CHMs, it is necessary to identify the storage time of CHMs according to the odor information using an E-nose system [113,114]. Wu [115] employed an E-nose system to identify different harvest periods of *Amomum villosum *Lour.*s* and distinguish their storage time (1 year and 2 years) based on the characteristic odor. Some analysis methods, PCA, LDA and PCA + LDA were employed to analyze the data. In PCA + LDA analysis model, PCA was used to select the odor feature subset and LDA was used for analyze and identification. The fitting correlation coefficients (R^2^) and root mean square error (RMSE) were calculated and treated as the criteria to evaluate the performance of these three models. The final results indicated that the identification performance of PCA + LDA (R^2^ = 0.9472, RMSE = 0.7618) was better than PCA (R^2^ = 0.9262, RMSE=0.8238) and LDA (R^2^ = 0.9086, RMSE = 0.8952). Zou [116] detected the odor of *Panax quinquefolium *L. (Xiyangshen) after different storage times (one year and three years) and established a discrimination model based on an E-nose. The performance of the proposed model was evaluated by ten-fold cross validation and external test sets while the sensor array was optimized by stepwise discriminant analysis. The identification rate of 89.76% with ten-fold cross validation demonstrated that the E-nose system could successfully identify *Panax quinquefolium *L. samples with different storage times. In Table 6, a summary of reviewed articles related to the application of E-nose systems for identification and classification of CHMs is presented.

## 5. Discussion: Challenges and Future Perspectives

### 5.1. Challenges

#### 5.1.1. Qualitative Analysis of VOCs in CHMs

In this paper, we have outlined major contributions of E-nose technologies as applied to the identification of CHMs by reviewing the most related and recent published articles (Table 6). As a result of this literature review, we have found that each paper focuses on a particular aspect of E-nose as applied to the identification of CHMs. Most of the publications present limited feasibility studies with poor validation, especially in terms of reproducibility and predictability [117,118]. Moreover, most of the reported results are based on limited experiments without proposing a specific identification process in the field of CHMs. While, the applications of E-nose technology in food, environmental monitoring, security and clinical diagnostics has been growing fast, on the other hand, in the field of CHMs, the applications of E-nose system have been limited and therefore, there is room for more research. Moreover, it was found that the composition of each kind of CHMs is very complex and often contains a variety of odors with different strengths. An E-nose system only obtains the overall odor of the sample and provides only a qualitative analysis of the volatile components in CHMs. Therefore, it is necessary to study the functionality of E-nose systems in order to achieve better chemical compositional analyses of volatile chemicals.

#### 5.1.2. Development of New Sensor Materials

The repeatability and stability of the sensor array in E-nose systems are easily influenced by environmental factors (such as humidity, temperature and vibration), which lead to the instability of the measurement data. In addition, the number of gas sensors in an E-nose system is limited and each gas sensor is usually sensitive to one kind of odor, so it is crucial to identify more sensitive, selective and stable sensing materials to construct the sensor arrays. Different devices such as SAW, molecularly imprinted polymer sensors [44], nano-bioelectronics [42], optical sensors [119], mobilized natural receptor sensors [120] and electrochemical biosensors [43,121] have shown suitable characteristics due to their compact structures, high sensitivity and fast response time [44,119]. In addition, ion mobility spectrum (IMS) [120,122] and organic printable electronic materials [45,48,123] with advantages of high sensitivity, simple apparatus, small volume and low cost, have shown great potential in standard E-nose technology, so the second challenge for E-nose technology is to investigate new materials and make it more portable and more sensitive with faster response times when exposed to different volatile species.

#### 5.1.3. Investigation of Appropriate Pattern Recognition Methods

The pattern recognition methods adopted in an E-nose system for the identification of CHMs, lack standard procedures, and provide unsatisfactory recognition accuracy. In Europe, there was an attempt to standardize the pattern recognition system [47]. Although differences in odor can be measured by an E-nose system, there is not full agreement on adopting a specific parent recognition method as applied to a particular sensory application. Moreover, much research needs to be done to achieve better interpretation and analysis of the results of identification of CHMs by an intuitive way. Therefore, another research challenge in this field is to investigate more appropriate pattern recognition methods in relation to the E-nose technology.

### 5.2. Future Perspectives

#### 5.2.1. Development of New Drugs

In addition to the identification and prediction of the kinds and quality of CHMs, the E-nose technology can also be useful in the development of new drugs and drug discovery [1,124,125,126]. A certain class of odor representing a group of chemicals may also relate to a certain class of drug. For example, some traditional Chinese medicines have “Ginseng flavor” or “Pungent flavor”, which represent tonic or dampness medicines. Therefore, during the development of new drugs, there is a chance to discover new medical value according to the detected odor and classification results using novel E-nose systems [126,127].

#### 5.2.2. Odor Standardization for CHMs

Up to now, odor still lacks a standardized format, and it is difficult to transfer and communicate odor to other users, especially when they are in remote places [47]. This has limited the development of a standardization approach for E-nose systems as applied to quality control of CHMs. In order to study a standard method for odor identification, scientists have tried to digitally describe odor by using smell codes. They have attempted to verbally describe odors based on perception and assess odor qualities by perception-based ratings. More systematic experimental trials were conducted, such as chemical structure-based and perception-based methods that have been used to classify and quantify odors [127,128]. Multiple component metrics of odor structure were used for beef flavor description [129]. However, all of these approaches are still in preliminary stages and to the best of our knowledge, there is no device that objectively expresses odor-related information. Therefore, it is necessary to develop novel experimental platforms with higher sensitivity and processing speed for odor standardization.

A novel and portable E-nose system with advanced features such as low cost, high sensitivity and fast response time can be employed for coding odor information and identifying primary odor molecules of CHMs. This multiplexed E-nose system, as an emerging tool for odor standardization, can generate combinatorial patterns of odors in order to serve as a universal code for odors that can be standardized for coding many odors [42,126,127]. The realization of odor standardization offers great potential for CHMs in many applications. It is expected to accelerate odor identification and quality control standardization for CHMs by providing codified odors.

#### 5.2.3. Odor Remote Reproduction and Remote Diagnosis in Medicine

In the future, with the realization of odor standardization, the subjective information of smell can be converted into objective information and even can be transferred to other remote places via the Internet. Like vision and hearing which have being encoded or decoded by related devices, smell obtained from odorant detection can be recognized and encoded into a standard format by the sensory system. Then, these standardized odor information can be analyzed by a data processing system, and transmitted to the remote olfactory display system. Decoding the odor information can be utilized in the reproduction of the odor based on an odor–receptor interaction database. Therefore, users in remote area would be able to smell the same odor as doctors for diagnosing those remote patients.

## 6. Conclusions

The E-nose technology can be employed to digitalize the odor information of CHMs and make it objective for qualitative analysis. Although, E-noses have been widely used in many applications there is limited literature on the application of E-nose systems in the field of CHMs. This might be due to the fact that the composition of each kind of CHMs is very complex and often contains a variety of odors with different strengths. However, there has been some progress in recent years in this area but there are more opportunities for further research and development to answer some fundamental research questions such as: (1) How to realize the qualitative analysis of volatile components and quality control in CHMs? (2) How to exploit new high-sensitivity sensor materials? (3) How to design highly accurate pattern recognition methods? (4) How to accomplish odor standardization? These challenges will be accompanied with the development of E-nose technology. We have found that through continuous improvement and development of E-nose technology, odor may become a new quantitative index for CHM quality control, which will advance further study of CHMs and the development of new drugs. At the same time, odor standardization and odor remote reproduction based on novel E-nose systems, will promote the objective and standardized development of Traditional Chinese Medicine.

## Figures and Tables

**Table 1:** E-nose models for commercial and non-commercial applications with different sensors such as Metal Oxide Sensor (MOS) and Conducting Polymer (CP).

State	Model	Number of Sensors	Sensor Material	Manufacturer	Country
Commercial	i-Pen, i-Pen3, PEN3	6, 10	MOS	Airsense Analytics	Germany
	Artinose	38	MOS	Sysca AG	Germany
	Air quality module	2	MOS	Applied Sensor	Sweden
	Aromascan A32S	32	CP	Osmetech Plc	USA
	Bloodhound ST 214	14	CP	Scensive Technologies	UK
	Cyranose 320	32	CP	Sensigent	USA
	FOX 3000, 4000	12, 18	MOS	Alpha MOS	France
	LibraNose	8	Quartz Crystal Microbalance (QCM)	Technobiochip	Italy
	iNose, T-nose	14, 10	MOS	Isenso	China
Non-commercial	Bioelectronic noses	--	Olfactory receptors	Ref [42]	--
	Molecularly imprinted polymers noses	--	Molecularly imprinted polymers	Ref [43,44,45]	--
	Optical sensors	--	Optical material	Ref [42,46,47]	--
	Nano-bioelectronics	--	Nanomaterials, animal receptors	Ref [48,49]	--

**Table 2:** Comparison of characteristics of sensors utilized in design of E-noses (sensor matrixes).

Sensor Type	Working Principle	Advantages	Disadvantages
Electrochemical sensors (EC)	The sensor reacts with the gas and generates an electrical signal proportional to the gas concentration gas.	1. Low power consumption 2. Good robustness 3. Room temperature operation	1. It isn’t applicable to aromatic hydrocarbons 2. Low sensitivity 3. Large volume
Metal oxide sensors (MOS)	The surface gas and oxide react to generate resistance changes according to the gas concentration.	1. Fast response, short recovery 2. High sensitivity 3. Long life, High reproducibility, convenient replacement	1. It is easy to react with sulfur compounds and produce damage to the sensor 2. Work at high temperature, High power consumption
Conducting polymer sensor (CP)	The resistance of the sensor is changed by the chemical reaction between the surface gas and the polymer, which forms the electrical signal.	1. High sensitivity 2. Fast response, short recovery 3. Easy synthesis 4. Room temperature operation 5. Not easy to corrosion by sulfur compounds or weak acids	1. Sensitive to environmental humidity 2.complex manufacturing process 3. Sensor life is short, generally 9~18 months
Surface acoustic wave sensors (SAW)	The surface gas flows through the sensors consisting of piezoelectric material and adsorbing material, which generates surface wave.	1. Fast response 2. Low cost 3. Miniaturization	1. High power consumption, high signal to noise ratio 2.Complex manufacturing process 3. Interface circuit complexity
Optical sensors (OS)	Measure the modulation of light properties or characteristics, such as changes in light absorbance, color, wave-length (colorimetric), upon exposure to gas analytics.	1. Low energy consumption 2. High signal-to-noise ratio 3. High sensitivity	1. Poor adaptability to environment 2. Low accuracy when long distance measurement
Biomimetic sensors (BS)	Sensors are composed of a fixed cell, an enzyme or other bioactive substances.	1. Good performance 2. High sensitivity 3. Suitable for on-site analysis 4. Suitable for more complex applications	1. Poor repeatability 2. Poor stability 3. Difficult to mass production

**Table 3:** Methods of data preprocessing for baseline correction.

Methods	Formula
Difference	$X_{ij}\left(t\right)=\left|S_{ij}\left(t\right)-S_{j}\left(0\right)\right|$
Relative	Xij(t)=Sij(t)Sj(0)
Fraction	Xij(t)=|Sij(t)−Sj(0)|Sj(0)
Sensor auto scaling	Xij(t)=(Sij(t)−sijminj(t))(Sijmaxj(t)−minj(t))
Array Auto Scaling	Xij(t)=Sij(t)(1n∑iSij2)12

Notes: *n* is the dimension of the sensor array; *p* is the number of test samples; $S_{j}\left(0\right)\left(j=1\sim n\right)$ expresses the output of the* j*th sensor in the initial state, that is, the baseline value of the sensor; $S_{ij}\left(t\right)\left(i=1\sim p,\;j=1\sim n\right)$ expresses the output value of the *j*th sensor of the *i*th test sample at the time of *t*.* X_ij_*(*t*) (*i = *1~*p*,* j* = 1~*n*) expresses the value of baseline correction.

**Table 4:** Methods of data preprocessing for data transformation.

Methods	Formula
Logarithmic	$X_{ij}\left(t\right)=\log\left(\left|S_{ij}\left(t\right)-S_{j}\left(0\right)\right|\right)$
First derivatives	$X_{ij}\left(t\right)=S_{ij}\left(t\right)-S_{ij}\left(t-1\right)$
Second derivatives	$X_{ij}\left(t\right)=\left(s_{ij}\left(t+1\right)-s_{ij}\left(t\right)\right)-\left(s_{ij}\left(t\right)-S_{ij}\left(t-1\right)\right)$

Notes: *n* is the dimension of the sensor array; *p* is the number of test samples; $S_{j}\left(0\right)\left(j=1\sim n\right)$ expresses the output of the* j*th sensor in the initial state, that is, the baseline value of the sensor; $S_{ij}\left(t\right)\left(i=1\sim p,\;j=1\sim n\right)$ expresses the output value of the *j*th sensor of the *i*th test sample at the time of *t*.* X_ij_*(*t*)* (i* = 1~*p*, *j* = 1~*n*) expresses the value of baseline correction.

**Table 5:** Common pattern recognition methods for E-nose systems.

Model	Common Method	Basic Principle	Application Area
Statistical recognition model	Principal component analysis (PCA)	A mathematical statistical analysis method. A set of related variables are converted to another set of linear unrelated variables by orthogonal transformation, and the linear unrelated variables are called principal components.	Medical information classification, population statistics, mathematical analysis.
	Linear discriminant analysis (LDA)	The high dimensional sample data is projected into a low dimensional vector space, which is conducive to the best classification. So in the new subspace, there is a greater distance between the class and a smaller distance in class.	Face recognition, identification of CHMs.
	Support vector machine (SVM)	It is based on statistical learning theory including two basic principles, VC (Vapnik-Chervonenkis) dimension theory and structural risk minimization principle. It shows many unique advantages in solving small samples, nonlinear and high dimensional pattern recognition.	Biological information processing, text classification and handwriting recognition.
	K-nearest neighbor (KNN)	It is to determine the classification of the samples according to the nearest one or a few samples. The algorithm is simple and easy to implement, and especially is suitable for multiple classification problems.	Forecast estimate, biological, medical, economic and other fields.
Intelligent recognition model	Artificial neural network (ANN)	By imitating the behavior characteristics of human or animal neural network, a mathematical model is established which is to carry out the distributed information processing.	Pattern recognition, intelligent robot, automatic control, prediction and estimation, biology, medicine, economy, etc.
	Deep learning (DL)	The feature of the original space is transformed into the feature of the new space, and the hierarchical feature representation is obtained by the multilayer feature transform.	Speech recognition, synthesis and Machine Translation; image classification and recognition, etc.
	Fuzzy inference (FIS)	Based on the fuzzy set theory, the method is to simulate the human brain to process the non-accurate or nonlinear data information.	Household electrical appliances, expert system, intelligent control, etc.
	Genetic algorithm (GA)	The method is to simulate the process of natural evolution and to search for the optimal solution, which consists of selection operation, exchange operation and mutation operation.	Function optimization; production scheduling problem, automatic control, image recognition, etc.

**Table 6:** Main applications of E-nose systems for CHMs identification and classification.

Selected Samples	Experimental Results	E-Nose Model	Data Processing Algorithm	Ref.
*Pogostemon cablin *(*Blanco*) Benth.,* Mentha haplocalyx *Briq	The correct recognition rates were 100% (LDA model) and 98% (PCA model)	PEN3 (Airsense Analytics, Germany)	PCA, LDA	[91] Liu, H.X.
Six kinds of *Zanthoxylum bungeanum *Maxim	BP-NN analysis was the best among three selected methods, and the initial discriminant rate and cross validation rate in BP-NN analysis were 99% and 96.2% respectively.	E-nose System (made up of eight sensors constructed in Lab)	Back Propagation Neural Network (BP-NN), Probabilistic Neural Network (PNN), SVM	[92] Wu, L.L.
*Apiaceae* plants	The identification rate of ten-folds cross validation was 94.71%.	FOX3000 (Alpha MOS, France)	LDA, PCA, Hierarchical clustering analysis (HCA), ANN	[93] Lin, H.
Seven medicines (*Illicium verum *Hook. f., *Amomi Fructus Rotundus*, *Ligusticum chuanxiong *hort., *Eugenia caryophyllata *Thunb., *Schizonepeta tenuifolia *Briq., *Cinnamomum cassia *Presl, *Amomum villosum *Lour.)	The correct recognition rates were 98% (LDA model) and 96% (PCA model) respectively.	PEN3	LDA, PCA	[94] Liu, H.X.
*Amomum villosum *Lour., *Pogostemon cablin *Benth., *Leonurus japonicus *Houtt., *Houttuynia cordata *Thunb., *Mentha haplocalyx *Briq. and *Bupleurum chinense *DC.	The odor fingerprint of *Mentha haplocalyx *Briq. had 15 common peaks and the largest average value, while that of *Bupleurum chinense *DC. had only 11 common peaks and the smallest average value.	PEN3	LDA, PCA, LDA + PCA	[95] Luo, D.
Raw *Atractylodes macrocephala *Koidz. and processed *Atractylodes macrocephala *Koidz	The RSD of the relative peak area of the common peaks were less than 1.2%, and the relative retention time of each peak was less than 1.1%.	FOX 3000	PCA	[97] Shen, G.
Four different samples of processed *Coptis chinensis *Franch.	PCA analysis was the best one in the selected four methods, and the initial discriminant rate and cross validation rate in PCA analysis were 100% and 94.4% respectively.	FOX 4000 (Alpha MOS, France)	PCA, LDA, Statistical Quality Control analysis (SQC), Soft Independent Modeling analysis (SIMCA)	[98] Xu, M.
Raw *Areca catechu *L. and processed *Areca catechu *L.	ANN analysis showed the best performance among three selected methods, and the initial discriminant rate and cross validation rate in ANN model were 100% and 97% respectively.	FOX 4000	PCA, LDA, ANN	[99] Huang, X.S.
*Siegesbeckia orientalis *L. from different producing areas	The ten-folds cross validation rate was 93.19%.	FOX 3000	PCA	[100] Kong, F.Y.
*Leonurus japonicus *Houtt. from Sichuan	PCA showed better performance than DFA.	FOX 4000	PCA, DFA	[101] Zhong, L.
*Fritillaria cirrhosa *D. Don and* Fritillaria thunbergii *Miq	The initial discriminant rate and cross validation rate were 98% and 95% respectively.	FOX 4000	PCA	[102] Wu, N.
Chinese *Panax ginseng *C.A. Mey. and Korean* Panax ginseng *C.A. Mey	The ten-folds cross validation rates of the three models were 96.12%, 97.56%, 92.39% respectively.	FOX 3000	PCA, Discriminant factorial analysis (DFA), SIMCA (soft independent model of class analogy)	[103] Li, S.
*Ligusticum chuanxiong *hort. samples from different regions	The correct identification rate was 92.1% based an E-nose system.	FOX 4000	PCA, LDA	[104] Chen, L.
*Chrysanthemummorifolium* RaTnat. in different habitats	The cross validation rates were 94.38% for PCA and 91.46% for DFA.	FOX 4000	PCA, DFA	[105] Han, B.X.
Identification of *Mentha haplocalyx *Briq. from different regions; The odor fingerprint of *Mentha haplocalyx *Briq	Samples from Guangdong province had 18 common peaks with the average value of 12.67, while *Mentha haplocalyx *Briq. from Guangxi province had only 14 common peaks and the average value of 11.81.	PEN3	PCA, PLS	[106] Zheng, J.B.
*Amomum villosum *Lour. from different regions	The performance of NBN model was the best and the initial discriminant rate and cross validation rate were 98% and 95.2% respectively.	FOX 3000	PCA, Fisher-LDA, Naive Bayes Net (NBN), Radial Basis Function (RBF), Random Forests (RF)	[107] Zou, H.Q.
*Oxybaphus himalaicus *Edgew. from different regions	The performance of ANN model was the best and the initial discriminant rate and cross validation rate were 100% and 96.8% respectively.	FOX 3000	DFA, HCA, ANN	[108] Lin, H.
*Saposhnikovia divaricata* (Turcz.) Schischk., *Bupleurum Chinense *DC. and *Angelica sinensis *(Oliv.) Diels.	SIMCA had more advantages in identification of *Angelica sinensis *(Oliv.) Diels than the other two models. The IDR and 10-FCVR were 96.6% and 95.2%; in identification of *Bupleurum Chinense *DC., IDR and 10-FCVR were 94.8% and 93.9%; in identification of *Saposhnikovia divaricata* (Turcz.) Schischk., IDR and 10-FCVR were 91.8% and 88.3%.	FOX 3000	PCA, SIMCA, DFA	[109] Wang, W.T.
*Amomum villosum *Lour. in different storage times	The identification performance of PCA + LDA (R^2^ = 0.9472, RMSE = 0.7618) was better than PCA (R^2^ = 0.9262, RMSE = 0.8238) and LDA (R^2^ = 0.9086, RMSE = 0.8952).	PEN3	PCA, LDA, PCA + LDA	[115] Wu, S.Y.
*Panax quinquefolium *L. in different storage times	The identification rate 89.76% of ten-folds cross validation showed that the E-nose system could also identify *Panax quinquefolium *L. samples with different storage time.	FOX 3000	ANN	[116] Zou, H.Q.
